# Trends in urine sampling rates of general practice patients with suspected lower urinary tract infections in England, 2015–2022: a population-based study

**DOI:** 10.1136/bmjopen-2024-084485

**Published:** 2024-08-06

**Authors:** Laura Ciaccio, Holly Fountain, Elizabeth Beech, Colin S Brown, Alicia Demirjian, Sarah Gerver, Berit Muller-Pebody, Sabine Bou-Antoun

**Affiliations:** 1 Division of Population Health and Genomics, University of Dundee School of Medicine, Dundee, UK; 2 Healthcare-Associated Infection (HCAI), Fungal, Antimicrobial Resistance (AMR), Antimicrobial Use (AMU) & Sepsis Division, UK Health Security Agency, London, UK; 3 NHS England, Taunton, UK; 4 UKHSA and NIHR Health Protection Research Unit in Healthcare Associated Infections and Antimicrobial Resistance, Imperial College London, London, UK; 5 Department of Paediatric Infectious Diseases & Immunology, Evelina London Children's Hospital, London, UK; 6 Faculty of Life Sciences & Medicine, King’s College London, London, UK

**Keywords:** EPIDEMIOLOGY, Primary Care, Public health, Urinary tract infections

## Abstract

**Abstract:**

**Objectives:**

Inappropriate prescribing of antibiotics is a key driver of antimicrobial resistance. This study aimed to describe urine sampling rates and antibiotic prescribing for patients with lower urinary tract infections (UTIs) in English general practice.

**Design:**

A retrospective population-based study using administrative data.

**Setting:**

IQVIA Medical Research Database (IMRD) data from general practices in England, 2015–2022.

**Participants:**

Patients who have consulted with an uncomplicated UTI in England general practices captured in the IMRD.

**Outcome measures:**

Trends in UTI episodes (episodes were defined as UTI diagnosis codes occurring within 14 days of each other), testing and antibiotic prescribing on the same day as initial UTI consultation were assessed from January 2015 to December 2022. Associations, using univariate and multivariate logistic regressions, were examined between consultation and demographic factors on the odds of a urine test.

**Results:**

There were 743 350 UTI episodes; 50.8% had a urine test. Testing rates fluctuated with an upward trend and large decline in 2020. Same-day UTI antibiotic prescribing occurred in 78.2% of episodes. In multivariate modelling, factors found to decrease odds of a urine test included age ≥85 years (0.83, 95% CI 0.82 to 0.84), consultation type (remote vs face to face, 0.45, 95% CI 0.45 to 0.46), episodes in London compared with the South (0.74, 95% CI 0.72 to 0.75) and increasing practice size (0.77, 95% CI 0.76 to 0.78). Odds of urine tests increased in males (OR 1.11, 95% CI 1.10 to 1.13), for those episodes without a same-day UTI antibiotic (1.10, 95% CI 1.04 to 1.16) for episodes for those with higher deprivation status (Indices of Multiple Deprivation 8 vs 1, 1.51, 95% CI 1.48 to 1.54). Compared with 2015, 2016–2019 saw increased odds of testing while 2020 and 2021 saw decreases, with 2022 showing increased odds.

**Conclusion:**

Urine testing for UTI in general practice in England showed an upward trend, with same-day antibiotic prescribing remaining consistent, suggesting greater alignment to national guidelines. The COVID-19 pandemic impacted testing rates, though as of 2022, they began to recover.

**STRENGTHS AND LIMITATIONS OF THIS STUDY**

- This study used a large population data set with established generalisability over a long study period (2015–2022) across general practice in England.
- The longitudinal, patient-level data set used allows for the identification of patients who consulted with a lower urinary tract infection and the linking of multiple datasets over time.
- The data used were collected for clinical use and not for research purposes, which may limit the accuracy of the urine sampling variable and may not show the true prevalence of all laboratory samples sent for analyses and may not capture all results from the samples sent to be cultured.
- Susceptibility results were not available for this study, meaning that appropriateness could not be assessed for patients with antibiotic prescriptions.

## Introduction

 In recent years, there has been little work nationally to assess urinary tract infections (UTIs), urine sampling rates and antibiotic prescribing.^1^ However, it is known that the majority of all antibiotic prescribing occurs in general practice (GP)^2^ and that UTIs are the second most common indication for antibiotics in England.^3^ Increased antibiotic use, particularly inappropriate antibiotic prescribing, increases selection pressure on bacteria and can increase rates of antimicrobial resistance (AMR) in the population.^4^ In GP, it has been found that receiving an antibiotic can double the risk of resistant urinary and respiratory bacterial infections for up to a year.^5^

Urine sampling with culture allows for the identification of organisms causing infection and for susceptibility testing to occur, which aids in the appropriate treatment of UTIs and antimicrobial stewardship, improving treatment outcomes and reducing the risk of AMR. AMR is often associated with higher morbidity and mortality, as well as higher healthcare costs^1^; this increases with age, with the incidence of sepsis as a result of UTI and its associated mortality increasing disproportionately.^6^ Looking at urine sampling and antibiotic prescribing on a patient level is, therefore, beneficial as it allows for these associations to be assessed by subgroups such as age and sex.

In the UK, the National Institute for Health and Care Excellence (NICE) guidelines provide testing and treatment pathway recommendations for lower UTIs based on four main patient groupings: pregnant women over 12 years, non-pregnant women aged 16 years and older, men aged 16 years and older, and children under 16 years.^7^ For each group, recommendations for testing vary; testing is not as highly emphasised for non-pregnant women, with a midstream urine culture recommended for pregnant women and men, with urine culture or dipstick advised for children aged under 16 years.^7^ Additional recommendations indicate that urine cultures should be sent for those over 65 years who are symptomatic and have been given an antibiotic, suspected pyelonephritis or sepsis cases, failed antibiotic treatment, recurrent UTI, catheterised patients prescribed an antibiotic or in those at higher risk of antibiotic resistance.^8^ The antibiotics recommended by the NICE guidelines include first-line choices of nitrofurantoin and trimethoprim, with only nitrofurantoin recommended for pregnant women. Second-line medications vary and include fosfomycin, pivmecillinam, amoxicillin and cefalexin.^7^ Though these antibiotics are specified in the lower UTI guidelines, other common antibiotics may be used for UTIs based on factors such as laboratory results or medical history.^9^ Recommended treatment lengths vary, with most recommending a 3-day course or up to 7 days in older populations and pregnant women.^10^ For some common UTI antibiotics, up to a 14-day treatment regimen is still recommended for select patient groups.^11^,^14^ Changes in NICE guidelines for uncomplicated lower UTIs,^7^ along with Quality Premiums offering financial incentives to encourage adherence to these guidelines^15^ and the publication of the 2019–2024 UK National Action Plan (NAP) on AMR^16^ have occurred in recent years, with no recent studies assessing differences in testing around these changes.

This study aims to assess changes in the occurrence of urine sampling practice and same-day antibiotic prescribing in GP for patients with a suspected lower UTI in the context of the 2018 NICE guidelines as well as the COVID-19 pandemic. Trends in the number of specimens with a positive result recorded will also be examined. The last time a similar analysis was completed at the national level included data captured up to 2015.^1^ This study looks to further help with ongoing work in understanding whether UTIs are being appropriately treated with antibiotics and if more can be done to help slow the development of resistance to the key antibiotics used for the treatment of UTIs.

## Methods

### Data source and management

Data used for this work were obtained from the IQVIA Medical Research Database (IMRD) from January 2015 to December 2022, inclusive. The IMRD captures longitudinal non-identified patient electronic healthcare records collected from patients of all ages (>5.4 million) from a sample of GP practices using IQVIA-compatible practice management systems (this includes data from practices using EMIS Health and Vision) in England. The IMRD data have been shown to be representative of the general population in England in terms of geography, age and sex.^17 18^ IMRD is non-identified data, with reference IDs able to be linked across different data tables. SNOMED and Read codes were matched to IMRD-specific clinical codes to identify lower UTI diagnoses in order to create the study cohort with these codes also used to identify urine culture and dipstick tests. All drug code IDs for prescriptions with the generic drug name being one of the antibiotics included in the 2018 lower UTI NICE guidelines (nitrofurantoin, trimethoprim, cefalexin, amoxicillin, fosfomycin and pivmecillinam) were pulled from the prescribing file. These antibiotic prescriptions were linked to UTI diagnosis codes and termed UTI antibiotics, though some are not exclusively used for the treatment of UTIs in practice. Additional descriptive analyses looked at all antibiotic prescriptions on the same day as the initial UTI consultation. For this analysis, all drug code IDs for those antibiotics with an Anatomical Therapeutic Chemical Classification System code of J01 (antibacterials for systemic use) and select additional alimentary tract and nitroimidazole derivatives for protozoal disease were included. Any data for the same patient that occurred on the same day and with the same diagnosis or drug code ID were considered deduplicates.

UTI episodes were created to account for multiple UTI consultations within the same episode of infection. UTI episodes were defined as any UTI diagnosis code occurring within 14 days of each other based on treatment length recommendations (online supplemental figure S1). For each UTI diagnosis code, any urine culture or dipstick that occurred within 3 days was linked. Once testing codes were linked, UTI diagnoses occurring within 14 days of each other were merged to create the UTI episodes. Any codes identified via clinical review for a positive urine culture or dipstick found within 14 days of a UTI diagnosis code were identified to calculate positivity rates.

For the UTI antibiotics, once episodes with a UTI antibiotic prescription were identified, same-day prescriptions were pulled for those episodes where the prescription date was the same as the first UTI consultation.

Following the linkage of UTI episodes, demographic data were linked to each episode. Variables included year of birth (from which age at the time of episode was calculated), sex, Indices of Multiple Deprivation (IMD) decile and predefined region (London, South, North and East Midlands).

### Statistical analysis

Once UTI episodes were fully linked, frequencies were run for a range of variables, including the number of episodes per month and the number of episodes per month with a urine culture, dipstick, UTI antibiotic and combinations of these variables. Trends were reviewed monthly, with annual frequencies calculated and tabled for comparison. These frequencies were further broken down by age group (<16, 16–44, 45–54, 55–64, 65–74, 75–84 and >85 years) and sex. To better contextualise these numbers, all frequencies were then calculated as percentages of all episodes, and for those broken down into age and sex, by the percentage of the population with an episode. Population counts were generated from the patient demography file provided in the IMRD data, with the number of patients registered with a GP calculated per year. All patient episodes were assigned to one of the four groupings in the NICE guidelines based on age, sex and pregnancy status. Based on the available data, pregnancy was identified using UTI pregnancy IMRD clinical codes. This is likely an underestimation of the prevalence of pregnancy in the cohort, and because of this, episodes including these pregnancy codes were removed from the final modelling. NICE groups were also used to identify episodes that received a first-line or second-line same-day UTI antibiotic or a combination of both.

In addition to the demographic characteristics, variables were included to characterise healthcare factors associated with UTI episodes. The type of episode was pulled from the consultation file and categorised as face to face, remote or other for those that did not fit either. GP practices were split into two sets of quartiles. The first based on the number of UTI episodes captured across the study period, with 1 being the lowest number and 4 being the highest. The second measure split practices into quartiles based on the number of patients registered with the practice. The number of patients registered at each practice was calculated using the registration numbers as of 2019, as it was the midpoint of the study period.

Following the trend analyses, univariate and multivariate logistic regression were run for variables selected a priori based on characteristics of interest available in the data. Variables included in the models were age group, sex, IMD decile, if the episode had a same-day UTI antibiotic, choice of same-day antibiotics (none, first line only, second line only, another UTI-specific antibiotic (as defined by the NICE guidelines), an alternative antibiotic or any combination of the above), the episode type, the year of the episode, the region of the practice, the number of episodes by practice quartile, the size of the practice by quartile. Following univariate analyses, multivariate models were run, with the outcome of interest being any urine test (culture and/or dipstick), along with additional models looking at urine tests for those with a same-day UTI antibiotic and without a same-day UTI antibiotic.

All data analyses were completed by using RStudio V.4.1.2 with data pulled from Microsoft SQL Server Management Studio V.18.

### Patient and public involvement

None.

## Results

There were 978 007 UTI diagnosis codes captured between January 2015 and December 2022. After linking diagnosis codes within 14 days of one another, there were 743 350 UTI episodes for 252 205 patients, with the mean number of episodes per person being 2.18 for females and 1.18 for males. The number of UTI episodes remained consistent from 2015 to 2020, with a slight increase in episodes in 2020 compared with 2019 (table 1). This initial increase in UTI episodes in early 2020 was observed concurrently with a large uptick in the percentage of episodes which were remote compared with face to face (64.6% remote episodes in 2020 vs 25.6% remote episodes in 2019) as well as an increase in the percentage of episode for males (16.5% in 2019 vs 17.4% in 2020). Over the full study period, the majority of episodes (83.4%) were for females, with 16–44 years representing the largest age group (33.3%); however, the median age at episode was 54 years. The number of episodes per year remained consistent until 2021 and 2022 when the numbers decreased (table 1). The distribution of age groups remained similar between 2015 and 2019; however, there was a 7.3% increase in the 55–64 years group (11.0%–11.8%) and a 7.5% increase in the 75–84 years group (14.6%–15.7%) between 2019 and 2022, with a 4.5% decrease in 16–44 years (33.2%–31.7%) and a 5.2% decrease in episodes among those aged ≥85 years (11.5%–10.9%) over the same period.

**Table 1:** Episode demographics by year

Category	2015	2016	2017	2018	2019	2020	2021	2022
UTI episodes	93 581	96 996	96 033	95 780	93 343	95 263	89 103	83 251
Age group								
<16	6558(7.0%)	6839(7.1%)	6672(7.0%)	6618(6.9%)	6519(7.0%)	6221(6.5%)	6036(6.8%)	5912(7.1%)
16–44	32 746(35.5%)	33 421(34.5%)	32 130(33.5%)	31 840(33.2%)	30 961(33.2%)	31 366(32.9%)	28 702(32.2%)	26 416(31.7%)
45–54	9416(10.1%)	9804(10.1%)	9757(10.2%)	9635(10.1%)	9238(9.9%)	9630(10.1%)	8681(9.7%)	8300(10.0%)
55–64	9061(9.7%)	9682(10.0%)	9862(10.3%)	10 058(10.5%)	10 226(11.0%)	10 765(11.3%)	10 100(11.3%)	9828(11.8%)
65–74	11 782(12.6%)	12 102(12.5%)	12 271(12.8%)	12 311(12.9%)	12 037(12.9%)	12 036(12.6%)	11 334(12.7%)	10 646(12.8%)
75–84	13 100(14.5%)	13 700(14.1%)	13 814(14.4%)	13 913(14.5%)	13 619(14.6%)	13 922(14.6%)	13 752(15.4%)	13 042(15.7%)
>85	10 918(11.7%)	11 448(11.8%)	11 527(12.0%)	11 405(11.9%)	10 743(11.5%)	11 323(11.9%)	10 498(11.8%)	9107(10.9%)
Sex								
Male	14 471(15.5%)	15 522(16.0%)	15 411(16.0%)	15 766(16.5%)	15 427(16.5%)	16 560(17.4%)	15 585(17.5%)	14 479(17.4%)
Female	79 106(84.5%)	81 472(84.0%)	80 620(84.0%)	80 011(83.5%)	77 911(83.5%)	78 698(82.6%)	73 516(82.5%)	68 764(82.6%)
Deprivation status								
1	10 010(10.7%)	10 296(10.6%)	10 426(10.9%)	10 410(10.9%)	9424(10.1%)	9573(10.0%)	8758(9.8%)	8072(9.7%)
2	12 325(13.2%)	12 515(12.9%)	12 285(12.8%)	12 292(12.8%)	11 869(12.7%)	12 137(12.7%)	11 395(12.8%)	10 820(13.0%)
3	10 429(11.1%)	10 922(11.3%)	10 623(11.1%)	10 398(10.9%)	10 392(11.1%)	10 729(11.3%)	9845(11.0%)	9338(11.2%)
4	9880(10.6%)	10 417(10.7%)	9965(10.4%)	10 048(10.5%)	10 049(10.8%)	10 428(10.9%)	9905(11.1%)	9172(11.0%)
5	8765(9.4%)	8884(9.2%)	8615(9.0%)	8485(8.9%)	8272(8.9%)	8723(9.2%)	8146(9.1%)	7571(9.1%)
6	7498(8.0%)	7823(8.1%)	7683(8.0%)	7588(7.9%)	7254(7.8%)	7316(7.7%)	6737(7.6%)	6393(7.7%)
7	8541(9.1%)	8951(9.2%)	9122(9.5%)	9248(9.7%)	9080(9.7%)	9347(9.8%)	9007(10.1%)	8119(9.8%)
8	8841(9.4%)	9198(9.5%)	9231(9.6%)	9487(9.9%)	9207(9.9%)	9357(9.8%)	8578(9.6%)	8127(9.8%)
9	7070(7.6%)	7460(7.7%)	7328(7.6%)	7287(7.6%)	7189(7.7%)	7143(7.5%)	6821(7.7%)	6177(7.4%)
10	9855(10.5%)	10 130(10.4%)	10 362(10.8%)	10 187(10.6%)	10 319(11.1%)	10 258(10.8%)	9637(10.8%)	9249(11.1%)
NA	367(0.4%)	400(0.4%)	393(0.4%)	350(0.4%)	288(0.3%)	252(0.3%)	274(0.3%)	213(0.3%)
Region								
London	14 353(15.3%)	14 886(15.3%)	12 831(13.4%)	11 922(12.4%)	12 165(13.0%)	13 172(13.8%)	12 564(14.1%)	12 042(14.5%)
South	31 594(33.8%)	33 910(35.0%)	34 734(36.2%)	34 601(36.1%)	33 642(36.0%)	33 738(35.4%)	31 440(35.3%)	29 113(35.0%)
Midlands	29 011(31.0%)	29 087(30.0%)	28 425(29.6%)	28 848(30.1%)	27 674(29.6%)	27 956(29.3%)	26 982(30.3%)	25 251(30.3%)
North	18 623(19.9%)	19 113(19.7%)	20 043(20.9%)	20 409(21.3%)	19 862(21.3%)	20 387(21.4%)	18 117(20.3%)	16 845(10.2%)
NICE group								
Female, pregnant 12+	175(0.2%)	191(0.2%)	217(0.2%)	208(0.2%)	233(0.2%)	531(0.6%)	581(0.7%)	479(0.6%)
Female, 16+	73 518(78.6%)	75 673(78.0%)	74 899(78.0%)	74 310(77.6%)	72 306(77.5%)	73 134(76.8%)	68 089(76.4%)	63 546(76.3%)
Male, 16+	13 330(14.2%)	14 291(14.7%)	14 245(14.8%)	14 642(15.3%)	14 280(15.3%)	15 373(16.1%)	14 395(16.2%)	13 306(16.0%)
Children <16	6554(7.0%)	6839(7.1%)	6670(6.9%)	6617(6.9%)	6519(7.0%)	6220(6.5%)	6036(6.8%)	5912(7.1%)

NAnot availableNICENational Institute for Health and Care ExcellenceUTIurinary tract infection

Of the total UTI episodes, 42.2% had a urine culture, and 25.1% had a dipstick. However, these are not mutually exclusive and overall, 50.8% had any urine test (32.5% of those with a test had both test types recorded). Of all UTI episodes, only 8.5% had a positive urine culture result captured (20.1% of those with a urine sample sent for culture), and 18.3% had a positive dipstick (72.9% of those tested with a dipstick). Between 2015 and 2019, there were year-on-year increases in urine testing among UTI episodes (52.7% in 2015 to 55.9% in 2019); however, a 31% decrease was observed in 2020 compared with 2019, with only 39.0% of episodes having a recorded urine test. Slight increases were observed in 2021 and 2022, but the percentage of UTI episodes with a urine test recorded at the end of the study had not recovered to the peak percentage seen in 2019. A similar trend was observed for positivity rates, which decreased by 41.7% and 37.9%, respectively, for urine culture and dipstick tests between 2019 and 2020 (table 2).

**Table 2:** Urine tests and antibiotic prescribing counts and percentages of episodes per year

	2015	2016	2017	2018	2019	2020	2021	2022
Total number of episodes	93 581	96 996	96 033	95 780	93 343	95 263	89 103	83 251
Number of patients	70 415	73 135	71 879	71 435	69 693	70 402	66 507	62 518
With a urine culture	38 722 (41.4%)	41 606 (42.9%)	41 619 (43.3%)	43 425 (45.3%)	43 791 (46.9%)	32 628 (34.3%)	35 462 (39.8%)	36 099 (43.4%)
With a dipstick	24 536 (26.2%)	27 361 (28.2%)	27 990 (29.1%)	27 031 (28.2%)	26 696 (28.6%)	16 673 (17.5%)	17 676 (19.8%)	18 867 (22.7%)
With urine test*	49 273 (52.7%)	53 099 (54.7%)	52 869 (55.1%)	53 216 (55.6%)	52 744 (55.9%)	37 167 (39.0%)	38 922 (43.7%)	40 212 (48.3%)
With positive urine culture result†	7543 (8.1%)	8359 (8.6%)	8595 (9.0%)	9555 (10.0%)	10 364 (11.1%)	6044 (6.3%)	6347 (7.1%)	6261 (7.5%)
Positive urine culture for episodes with urine culture (%)	19.5%	20.1%	20.7%	22.0%	23.7%	18.5%	17.9%	17.3%
With positive dipstick result†	17 777 (19.0%)	19 836 (20.5%)	20 360 (21.2%)	19 689 (20.6%)	19 431 (20.8%)	12 049 (12.6%)	12 900 (14.5%)	14 142 (17.0%)
Positive dipstick for episodes with a dipstick (%)	72.5%	72.5%	72.7%	72.8%	72.8%	72.3%	73.0%	75.0%
With a same-day UTI antibiotic	72 784 (77.8%)	76 361 (78.7%)	74 728 (77.8%)	74 244 (77.5%)	72 163 (77.3%)	75 945 (79.7%)	69 364 (77.8%)	65 494 (78.7%)
With a same-day UTI antibiotic and urine test*	38 561 (41.2%)	42 093 (43.4%)	41 595 (43.3%)	41 775 (43.6%)	41 106 (44.0%)	27 790 (29.2%)	28 807 (32.3%)	30 706 (36.9%)
Same-day UTI antibiotic and urine test for episodes with urine test* (%)	53.0%	55.1%	55.7%	56.3%	57.0%	36.6%	41.5%	46.9%

* Urine culture and/or dipstick.

† Within 14 days of diagnosis.

UTIurinary tract infection

Overall, 581 063 (78.2%) of UTI episodes had a NICE-defined UTI antibiotic prescription captured on the same day as their initial UTI diagnosis. The most prescribed UTI antibiotic was nitrofurantoin. The percentage of overall episodes with a same-day UTI antibiotic prescription remained around 78% across the entire study period, with only a slight increase to 79.7% in 2020. This differs from the previously described trends in testing and positive test results in the study period. Because of this, the percentage of episodes with a urine test and same-day UTI antibiotic was between 41% and 44% until 2020 when this decreased to 29.2%, only recovering to 36.9% by 2022.

When examining all other antibiotics prescribed on the same day as the initial UTI diagnosis, the most common antibiotics were those often indicated as alternative choices for UTI treatment. There were 38 different antibiotics captured, with 35 998 episodes (4.8%) having a non-UTI-recommended antibiotic captured on the same day as their first UTI diagnosis. The most often prescribed non-UTI recommended antibiotic was amoxicillin/clavulanic acid, with 19 330 episodes having at least one prescription and the second most prescribed being ciprofloxacin at 11 805 episodes. Of those episodes with a same-day UTI antibiotic, <1% also had another non-UTI antibiotic prescribed.

**Figure 1:**
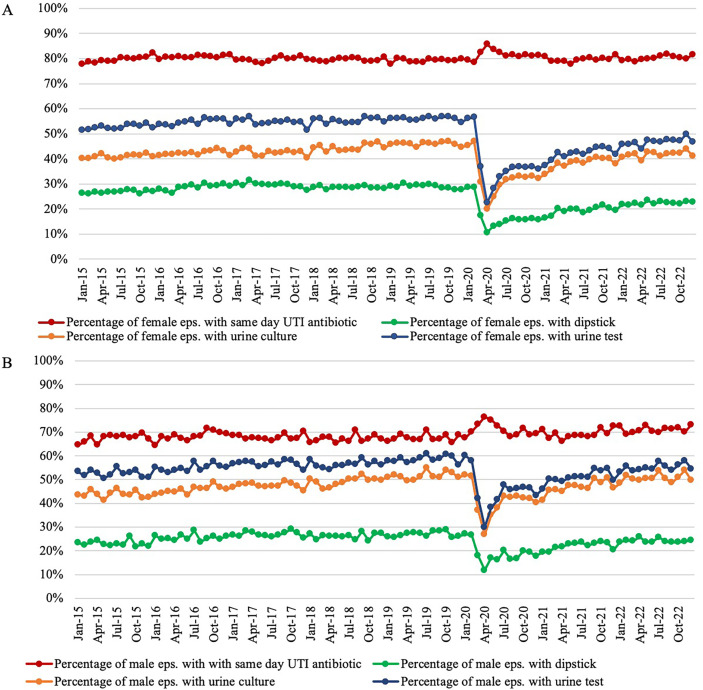
Percentage of episodes with urine testing and same-day UTI antibiotics for (**A**) females and (**B**) males. UTI, urinary tract infection.

When comparing UTI antibiotic prescribing and testing trends, the percentage of episodes with a UTI antibiotic prescription is consistently higher than the percentage with a urine culture or test (figure 1). The overall trend for the percentage of episodes with a UTI antibiotic remained steady between 2015 and 2019, with a slight increase in urine testing driven by urine cultures (online supplemental figure S2). However, the trend deviated in early 2020 at the start of the COVID-19 pandemic, where there was an increase in episodes with a UTI antibiotic (+5.6% from February to April 2020) and a large decrease in testing (−58.0% in urine tests from February to April 2020).

Overall, across the study period, the percentage of episodes with a positive result within 14 days of the first UTI diagnosis code was 20.1% for urine cultures (63 068/313 352) and 72.9% for dipsticks (136 184/186 830), respectively. There was a noticeable decrease of 36.6% in the percentage of positive urine cultures captured between July 2019 (954/3934 (24.3%)) and November 2020 (461/2989 (15.4%)). The rate of positive dipsticks remained consistent, with a slight increase seen from May 2021 onward. Of those episodes with a positive urine test result captured, 81.3% also had a same-day UTI antibiotic prescription; this peaked in 2016 (83.8%) and was lowest in 2021 (77.2%). Of those with a same-day UTI antibiotic, 23.7% also had a positive urine test, ranging from 15.6% in 2020 to 28.4% in 2019.

**Figure 2:**
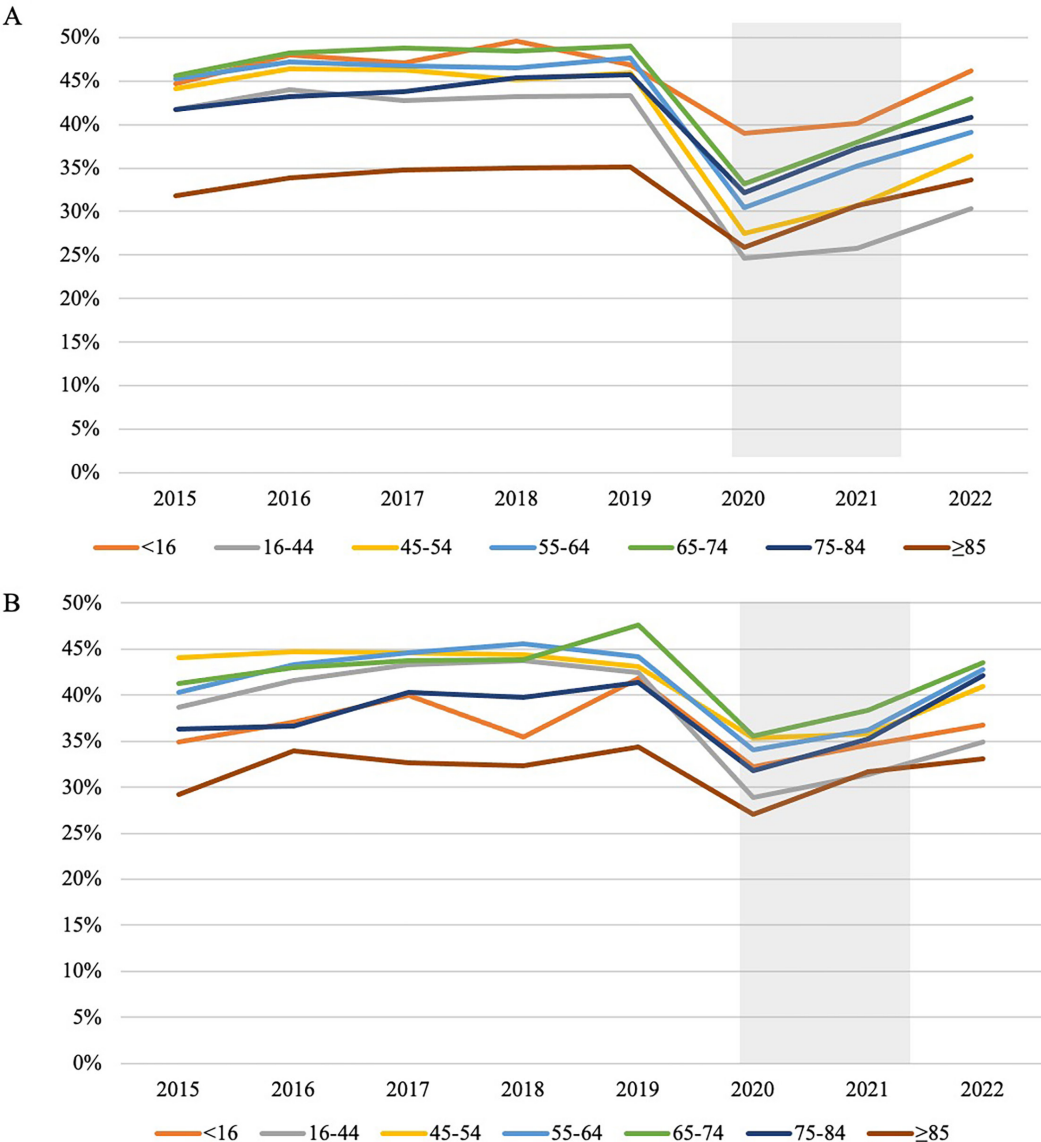
Percentage of episodes with a same-day UTI antibiotic prescription and urine test for (**A**) females, (**B**) males. UTI, urinary tract infection.

The percentage of UTI episodes where a same-day UTI antibiotic of interest was prescribed and a urine test was captured were examined by sex and age group. In total, 292 433 episodes (39.3%) had a same-day UTI antibiotic and urine test, with both females and males 65–74 years of age being the most often tested with a same-day UTI antibiotic. For both males and females, the percentage of UTI episodes with both a same-day UTI antibiotic and any urine test decreased in 2020 across all age groups, with increases observed in 2021 and 2022 compared with 2020. However, those females aged 16–84 years and males aged 16–54 years did not reach prepandemic levels by 2022 (figure 2).

Those ≥85 years showed a low rate of testing with a same-day UTI antibiotic. This is driven by testing, with a decrease of 25.7% of episodes with a urine test from 2019 (47.5% of episodes) to 2020 (35.3% of episodes) for females ≥85 years, and a decrease of 21.8% from 2019 (47.6% of episodes) to 2020 (37.2% of episodes) for males ≥85 years. This is in contrast to the 5.6% and 8.4% increase seen in same-day UTI antibiotic prescribing for females and males in this age group, respectively, over the same time period.

The rate of testing with a same-day UTI antibiotic prescription for those 16–44 years dropped 42.3% from 42.3% in 2019 to 25.0% in 2020. Similar to the ≥85 years group, this is driven largely by testing rather than prescribing, with urine testing decreasing for UTI episodes in this age group by 38.2% from 2019 (53.9% of all episodes in this age group) to 2020 (33.3% of all episodes in this age group) while same-day UTI antibiotic prescriptions increased 1.2% in the same period. Females aged under 16 years saw the smallest impact on testing and prescribing in 2020, with the highest level of testing and same-day prescribing (39.0%).

Prior to multivariate logistic regression modelling, episodes with a UTI in pregnancy code were removed. This was due to a low capture rate of pregnancy UTI codes in the cohort (0.4% of episodes), which led to removing episodes including these codes from the modelling. After these episodes were removed, 740 704 were used in the final models.

Multivariate logistic regression was performed to investigate the effect of risk factors on the odds of a UTI episode having a urine test, adjusted for other covariates (table 3). The factors found to have reduced odds of a urine test included remote consultations (vs face to face, OR 0.45, 95% CI 0.45 to 0.46), episodes in London when compared with those in the South (OR 0.74, 95% CI 0.72 to 0.75), episodes occurring within COVID-19 pandemic years (2020 and 2021, OR 0.78, 95% CI 0.76 to 0.79 and OR 0.98, 95% CI 0.96 to 0.99, respectively) and episodes at larger GP practices. Compared with episodes among those aged 16–44 years (the largest age group), all other age groups saw increased odds of testing, except notably among those ≥85 years (OR 0.83, 95% CI 0.82 to 0.84). Odds of urine test were increased for male episodes (OR 1.11, 95% CI 1.10 to 1.13), for those episodes without a same-day UTI antibiotic (OR 1.10, 95% CI 1.04 to 1.16) for those with higher deprivation status when compared with 1, particularly IMD 8 (OR 1.51, 95% CI 1.48 to 1.54), for those who received a same-day prescription for a non-UTI antibiotic (OR 1.06, 95% CI 1.03 to 1.09), in the north compared with the south (OR 1.01, 95% CI 0.99 to 1.02) and in practices with more UTI episodes, particularly those in quartile 3 compared with quartile 1 (OR 1.27, 95% CI 1.25 to 1.30).

**Table 3:** Associations between episode factors and the odds of a urine test (urine culture and/or dipstick) from binary logistic regression

Variable	Category	Frequency	Univariate OR(95% CI)	P value	Multivariable OR(95% CI)	P value
Age group	<16	51 367	1.72 (1.68 to 1.75)	<0.001	1.58 (1.55 to 1.62)	<0.001
	16–44	244 968	REF	–	–	–
	45–54	74 449	1.19 (1.17 to 1.21)	<0.001	1.15 (1.13 to 1.17)	<0.001
	55–64	79 578	1.31 (1.29 to 1.33)	<0.001	1.24 (1.22 to 1.26)	<0.001
	65–74	94 518	1.45 (1.42 to 1.47)	<0.001	1.33 (1.31 to 1.36)	<0.001
	75–84	108 857	1.28 (1.26 to 1.29)	<0.001	1.18 (1.16 to 1.20)	<0.001
	≥85	86 967	0.90 (0.88 to 0.91)	<0.001	0.83 (0.82 to 0.84)	<0.001
Sex	Female	617 483	REF	–	–	–
	Male	123 221	1.15 (1.14 to 1.17)	<0.001	1.11 (1.10 to 1.13)	<0.001
Deprivation status	1	76 624	REF	–	–	–
	2	95 164	0.96 (0.95 to 0.98)	<0.001	1.11 (1.08 to 1.13)	<0.001
	3	82 249	1.00 (0.98 to 1.02)	0.81	1.18 (1.15 to 1.20)	<0.001
	4	79 528	1.09 (1.07 to 1.11)	<0.001	1.27 (1.25 to 1.30)	<0.001
	5	67 193	1.09 (1.07 to 1.11)	<0.001	1.26 (1.23 to 1.29)	<0.001
	6	58 126	1.26 (1.23 to 1.28)	<0.001	1.41 (1.37 to 1.44)	<0.001
	7	71 188	1.27 (1.24 to 1.29)	<0.001	1.39 (1.36 to 1.42)	<0.001
	8	71 876	1.38 (1.36 to 1.41)	<0.001	1.51 (1.48 to 1.54)	<0.001
	9	56 367	1.26 (1.23 to 1.29)	<0.001	1.39 (1.36 to 1.43)	<0.001
	10	79 866	1.22 (1.19 to 1.24)	<0.001	1.32 (1.29 to 1.35)	<0.001
Same day UTI antibiotic	Yes	579 114	REF	–	–	–
	No	161 590	1.09 (1.08 to 1.10)	<0.001	1.10 (1.04 to 1.16)	<0.001
Choice of same-day antibiotic	None	129 061	REF	–	–	–
	First line	505 425	0.95 (0.94 to 0.96)	<0.001	0.96 (0.91 to 1.02)	0.16
	Second line	21 663	1.02 (0.99 to 1.04)	0.31	1.06 (0.99 to 1.13)	0.08
	Other UTI antibiotic*	46 387	0.99 (0.97 to 1.01)	0.27	1.03 (0.97 to 1.09)	0.30
	Other non-UTI-specific antibiotic	32 529	1.21 (1.18 to 1.24)	<0.001	1.06 (1.03 to 1.09)	<0.001
	Combination	5639	1.09 (1.03 to 1.15)	0.002	NA	<0.001
Consultation type	Face to face	411 135	REF	–	–	–
	Remote	280 626	0.43 (0.43 to 0.43)	<0.001	0.45 (0.45 to 0.46)	<0.001
	Other	32 083	0.33 (0.33 to 0.34)	<0.001	0.32 (0.31 to 0.33)	<0.001
Region	South	262 012	REF	–	–	–
	London	102 987	0.67 (0.66 to 0.68)	<0.001	0.74 (0.72 to 0.75)	<0.001
	Midlands	222 710	0.97 (0.96 to 0.98)	<0.001	0.91 (0.90 to 0.92)	0.22
	North	152 995	1.04 (1.03 to 1.06)	<0.001	1.01 (0.99 to 1.02)	<0.001
Year	2015	93 402	REF	–	–	–
	2016	96 803	1.09 (1.07 to 1.11)	<0.001	1.09 (1.07 to 1.11)	<0.001
	2017	95 841	1.10 (1.08 to 1.12)	<0.001	1.12 (1.10 to 1.15)	<0.001
	2018	95 569	1.12 (1.10 to 1.15)	<0.001	1.15 (1.12 to 1.17)	<0.001
	2019	93 105	1.17 (1.15 to 1.19)	<0.001	1.21 (1.18 to 1.23)	<0.001
	2020	94 727	0.58 (0.57 to 0.59)	<0.001	0.78 (0.76 to 0.79)	0.03
	2021	88 520	0.70 (0.69 to 0.71)	<0.001	0.98 (0.96 to 0.99)	<0.001
	2022	82 764	0.84 (0.83 to 0.86)	<0.001	1.10 (1.07 to 1.11)	<0.001
Episodes per practice quartiles	1 (lowest)	184 585	REF	–	–	–
	2	183 054	1.14 (1.12 to 1.15)	<0.001	1.24 (1.22 to 1.26)	<0.001
	3	190 061	1.15 (1.13 to 1.16)	<0.001	1.27 (1.25 to 1.30)	<0.001
	4	185 650	1.05 (1.03 to 1.06)	<0.001	1.21 (1.18 to 1.23)	<0.001
Practice size by quartiles	1 (smallest)	201 286	REF	–	–	–
	2	200 534	0.91 (0.90 to 0.92)	<0.001	0.83 (0.82 to 0.84)	0.55
	3	183 340	0.88 (0.87 to 0.89)	<0.001	0.77 (0.76 to 0.78)	<0.001
	4	158 826	0.92 (0.90 to 0.93)	<0.001	0.82 (0.80 to 0.84)	<0.001

* Includes UTI-specific antibiotics other than those included in the specified NICE guidelines for the patient based on age and sex.

NICENational Institute for Health and Care ExcellenceUTIurinary tract infection

The year of the episode also impacted the odds of testing when compared with 2015; 2016–2019 showed a year-on-year increase while 2020 and 2021 saw significant decreases in the odds of testing. However, 2022 saw testing odds increase again.

Additional sensitivity models were run for the same outcome: one for those episodes with a same-day UTI antibiotic and one for those without a same-day UTI antibiotic prescription captured. The multivariate model looking at those with a same-day UTI antibiotic showed the same trends as the primary model for all variables except the north no longer had increased odds of testing when compared with the south (OR 0.98, 95% CI 0.96 to 0.99). The model for episodes without a same-day UTI antibiotic prescribed had a few changes. The increased OR for those 75–84 in the primary model was no longer found to be statistically significant, and there was an increase in the odds of testing in all years for those without a same-day antibiotic. For all other variables, the overall trends aligned with the primary model (online supplemental table S1).

## Discussion

### Principal findings

This large patient-level study demonstrates the changes seen in overall testing and prescribing for patients with a suspected lower UTI in GP, with marked changes seen following the start of the COVID-19 pandemic. The results presented in this work provide updated data around testing and prescribing for lower UTIs in GP in England, including data prior to and following the introduction of the 2018 NICE lower UTI guidelines and the COVID-19 pandemic. This work is also able to build directly on previous work looking at testing and antibiotic prescribing.

Prior to the start of the pandemic, the trends in testing for UTI patients were slowly but continuously increasing. This increase in testing, with the highest levels seen in 2019, coincides with NICE guidelines published in late 2018, with a mid-stream urine sample recommended for susceptibility testing in pregnant women and men, with urine culture for susceptibility recommended for under 16s.^9 19^ This period also coincides with the publication of the 2019–2024 UK NAP on AMR in 2019, which included the aim of reporting on the percentage of antibiotic prescriptions supported by a diagnostic test or decision-support tool by 2024.^16^ However, following the start of the pandemic in 2020, testing became less likely due to changes in how care was able to be delivered due to COVID-19 restrictions and national lockdowns. This is seen in the change in the percentages of remote and face-to-face consultations in 2020, with remote consultations making up 25.6% of UTI episodes in 2019, up to 64.6% of UTI episodes in 2020 and 68.7% in 2021. The increase in remote UTI consultants was largest in those under 65, who in 2019 made up 55.6% of remote consultations, increasing to 61.0% in 2020. As the rate of remote consultations increased, so did rates of coding for ‘suspected UTI’, which made up 76.0% of the first diagnosis code captured for UTI episodes in 2019, up to 89.3% in 2020. Overall, urine testing in 2021 and 2022, though higher than the percentage seen in 2020, did not recover to prepandemic levels seen in 2018 and 2019. The rates of testing were found to have rebounded earlier for males rather than females, likely due to the emphasis on testing for men versus only for a subpopulation of females. The change in testing during the pandemic, with little change seen in antibiotic prescribing, likely means that empiric prescribing without confirmation of the infectious agent was common, which might impact downstream rates of AMR.

Same-day antibiotic prescribing was consistent across the study period, even when testing rates decreased. When testing in UTI episodes decreased by 38.0% from 2019 to 2020, same-day antibiotic prescribing increased by 3.1%. This rise in same-day prescribing in 2020 was likely due to an abundance of caution early in the pandemic when patients could not be seen face to face. This aligns with other work, which found that though overall community antibiotic prescribing declined early in the pandemic when the decrease in GP encounters is taken into account, there is a noted increase in the proportion of encounters prescribed an antibiotic.^20^

Nitrofurantoin and trimethoprim were the most commonly prescribed UTI antibiotics, particularly for non-pregnant individuals over 16, which aligns with the NICE guidelines.^9^ When looking at same-day antibiotic prescribing as a factor influencing the odds of testing, a same-day prescription was associated with lower odds of testing. This might have been due to non-pregnant, non-catheterised women with two or more UTI symptoms being prescribed an antibiotic without requiring urinary testing, as per testing guidelines.^8 9^ When univariate regression is used to examine odds of testing by NICE group, women ≥16 years had the lowest odds of testing compared with men ≥16 years (OR 1.18) and children (OR 1.56). This population and the treatment guidance have likely driven the results seen in the multivariate regression analysis.^9^ Though from 2015 to 2019, females had a higher percentage of episodes with a same-day antibiotic and test than men, the larger number of episodes for females is a contributing factor.

Due to the observed effects of the COVID-19 pandemic, it is difficult to fully determine the impact of the 2018 NICE guidelines, as these were only published at the end of 2018.^7^ Prior to these guidelines, there were no national UK guidelines for the treatment and management of lower UTIs in adults, with NICE publishing a UTI Quality Standard in 2015.^21^ The Quality Statements as part of this publication with relevance to the NICE guidelines include adults ≥65 years recommended to have a full clinical assessment to diagnose UTI, men with symptoms of an upper UTI referred for urological investigation, urine culture recommended for adults with a UTI not responding to antibiotic treatment, and antibiotics not recommended to treat asymptomatic bacteriuria in non-pregnant women.^21 22^

The impact of demographic factors, such as age and sex, on urine testing, aligns with previous literature and much of the NICE guidance.^1 7 9 23 24^ Age was found to increase the odds of testing compared with those 16–44, except in those ≥85. This aligns with the emphasis placed on both testing children with UTI symptoms found in the guidance, as well as the decreased emphasis on testing in much older adults due to frequent asymptomatic bacteriuria^1 9 24^ with urine culture testing often only performed in older adults with recurrent UTI or when resistance is suspected.^825^,^29^ The 2015 NICE Quality Standards also emphasised that the diagnosis of UTI is particularly difficult in older people due to asymptomatic bacteriuria. The prevalence of bacteriuria may be so high that urine culture ceases to be a reliable diagnostic test, with older people, particularly in long-term care, often receiving antibiotic treatment for asymptomatic bacteriuria despite evidence of adverse effects with little to no compensating clinical benefit.^22^ Based on the NICE guidance, it would also be expected that urine testing would be completed for the majority of men with suspected UTIs.^7^ While males in this study were shown to have higher rates of testing in GP (53.8%), testing in females was found to be only 6.5% less. Though pregnant women were likely included where UTI pregnancy codes were not accurately captured, which may have increased the overall rates, this is still higher than would be expected based on the guidelines.^7^ This may represent overtesting in this population, but without additional data regarding patient symptoms and comorbidities, confirming the appropriateness of testing in this group is difficult.

Deprivation status was also found to impact the odds of testing, with those in the least deprived groups having greater odds of testing. This aligns with prior work around healthcare provided by IMD deciles, though lower testing may be a concern as work in similar populations has found that those in the more deprived IMD groups are more likely to have more resistant infections as well as being more likely to receive a greater number of antibiotics.^30^,^33^

Remote episodes were also associated with reduced odds of testing compared with face-to-face episodes. Furthermore, following the start of the COVID-19 pandemic in 2020, the odds of testing decreased compared with 2019, which coincided with a change in GP consultation location, whereby in 2020, the majority of consultations were remote. Additional factors, such as those practices with more episodes having greater odds of testing, are likely due to the greater opportunity for testing in these practices. Episodes occurring in London were also found to have the lowest odds of urine testing. This is likely due to many factors, including fewer UTI episodes in London compared with all three other regions and an established lower rate of overall and UTI-specific antibiotic prescribing in London, which would also impact testing rates.^3 34^ As established in other work,^35^ antibiotic use is higher in secondary care settings, and patients often have greater access to secondary care and other GP settings (walk-in and urgent care centres) in London compared with other regions with this data unavailable in the data set used here.

### Comparison with other studies

Few recent studies have examined UTI testing and antibiotic prescribing, particularly following the 2018 NICE UTI guidelines. Many recent studies in this area have focused on UTIs in select populations rather than testing and prescribing across a broad sample of the population and often do not include the review of UTI testing and prescribing over time.^1036^,^39^ The patterns in UTI episodes found in this study align with overall patterns in UTI burden found in previous work.^1 37^ The decreased number of UTIs seen after the start of the pandemic aligns with similar work completed in other populations.^40^ This decrease also aligns with similar reductions in appointments for other common indications in primary care in England, such as respiratory infections.^18 41^

The results of this study build on the work of a previous study examining testing and prescribing for UTIs from 2011 to 2015, inclusive.^1^ This earlier study reported that a urine sample for culture or microscopy was requested within 10 days for 25% of UTI episodes, lower than the 42.2% within 3 days found in this work.^1^ However, it should be noted that in this study, a urine culture within 3 days of any UTI diagnosis code within a UTI episode was included, likely increasing the rate. This prior study also found that for this 10-day testing window, the percentage of women aged 18–64 years with a urine test was 30% lower than men of the same age group (23.6% vs 30.6%).^1^ The difference in testing is similar to the result presented here, with the difference in urine testing between males and females found to range from approximately 0.2% (51.6% males, 51.7% females in February 2015) up to 36% (46.6% for males, 35.0% for females in July 2020). From 2011 to 2015, men aged 18–64 years were shown to have the highest testing level at 30.6% of episodes, a lower rate than seen in this study for males aged 16–64 years (57.3%).^1^ However, in the current study, a greater percentage of females were also found to have a urine test (48.2% for females 16–64) compared with the 2011–2015 study (23.6% for women 18–64). The prior study also found that same-day antibiotics were prescribed in 85.7% of episodes, higher than the 78.2% found in this study. However, this work found a higher rate of urine tests and same-day antibiotics (39.3%) than the rate of antibiotic prescribing and urine testing from 2011 to 2015 (17%).^1^

Other existing studies examining the management of lower UTIs, including rates of testing and prescribing of antibiotics, have focused on older adults. One study looking at UTI incidence in older adults (>65 years) from 2004 to 2014 in UK primary care found an overall increase in UTIs across the study period in both men and women, with an increase in the use of UTI-specific antibiotics over the same period.^37^ This aligns with the increase seen in UTI episodes for males from 2015 to 2018 and in females from 2015 to 2017. While this study did not specifically look at antibiotic choice over time, the results did find that the percentage of UTI episodes with a same-day UTI-specific antibiotic prescription was lower (78.2%) compared with the 2004–2014 study (98%).^37^ Another study similarly looking at adults 65 and older with a community UTI diagnosis found that 87.3% of patients received an immediate antibiotic with men less likely to receive an immediate prescription.^25^ These results again align with the results found in this work, though overall same-day prescribing was slightly lower at 78.2%, with rates of same-day prescribing around 70.0% for males with females around 80.0%, with men possibly more likely to have delayed or withheld prescribing as found in the prior study.^25^

### Strengths and limitations

The primary strength of this study is the size and established generalisability of the data set used. Population trends aligned well with earlier work, allowing this study to further build on these previous analyses.^1 6^ Regarding the limitations of this work, though there was a large amount of data accessible for this study, the data used were collected for clinical use and not for research purposes. Therefore, some variables may not be well completed, as they are not needed in day-to-day clinical use. This may limit the accuracy of the urine sampling variable and may not show the true prevalence of every laboratory sampling sent for analysis. However, as previously mentioned, rates were found to be in range and aligned with other studies, lending greater confidence to the results.^1^ An associated limitation is that patient medical data is provided via GP records, and therefore, all the results from a sample being cultured may not be recorded if no observation/consultation occurred from the result or if urinalysis was conducted at a care setting outside of a GP practice. As part of this work, other indications and comorbidities besides lower UTI diagnoses were not examined in relation to the antibiotics prescribed. This limits the ability of this work to determine the appropriateness of the antibiotic prescriptions. By examining antibiotics commonly used to treat UTIs as specified in the NICE guidelines in conjunction with a UTI diagnosis on the same day, this study has tried to limit the analyses to those prescriptions most likely written for UTI treatment.

In addition, data for other clinical characteristics may be incomplete as well. Due to the structure of the data, it was difficult to determine pregnancy dates within the cohort. Though diagnosis codes for UTI in pregnancy were available and used for this work, using these codes alone likely does not accurately capture all pregnancies in the cohort. The rate of episodes with a UTI in pregnancy code that was able to be identified in this cohort was around 0.4%, while data from 2011 to 2015 looking at UTI diagnosis and treatment found <1% of UTI episodes were for pregnant women.^1^ It is also likely that many UTIs in pregnancy would not be captured in GP records, as many would seek care and be tested in midwifery-led clinics or at other prenatal services.

When examining UTI episodes, and, in particular, those with positive results captured within 14 days of a urine culture or dipstick, it should be noted that many of these may be asymptomatic bacteriuria rather than a UTI. This is particularly true for older populations where asymptomatic bacteriuria is more common and often leads to unnecessary testing and antibiotic prescribing.^26^,^2942^

Due to limitations in the diagnoses and symptom coding, we could not accurately capture if same-day prescriptions were designated as backup or delayed antibiotic prescriptions. Though these are part of the NICE guidance, with three READ/SNOMED codes designated by the Royal College of General Practitioners to capture data on these prescriptions,^43^ we found a very low rate of these codes in the data. In this study, there were no susceptibility results available for the urine cultures. This meant that appropriateness could not be fully determined for patients with multiple antibiotic prescriptions.

Regarding the methodology used, though binary logistic regression is a robust method for examining the odds of testing, an alternative approach using mixed effects models would have accounted for clustering by GP practice and patients with multiple episodes. This was unable to be completed within the project timeframe, however it is unlikely that the overall results would significantly change.

### Implication for policy and practice

The proportion of urine cultures with a result reported within the medical records in a timely manner may suggest the need for updated policies around test results being recorded in the patient’s central record. This would allow for easier and more efficient tracking of patient conditions and may help decrease the need for future testing. However, it should be noted that the percentage of positive urine culture captured in this work does fall within rates found in other work, which have been found to range from between 20% and 50%.^44^,^47^ The coding used for delayed prescribing was difficult to assess in this population despite the inclusion of delayed and backup prescribing in the NICE guidelines. This, again, could support future policies around coding and capturing delayed prescribing at the first encounter in medical records.

## Conclusions

Urine testing for suspected UTIs in GP in England showed an upward trend prepandemic, with same-day antibiotic prescribing remaining consistent across the study period. This may suggest greater alignment with national guidelines regarding testing over time. The COVID-19 pandemic drastically reduced the percentage of UTI episodes with a coded diagnostic test, though as of late 2022, this has begun to recover to prepandemic levels. Outside of short-term increases early in the pandemic, same-day UTI antibiotic prescribing has not increased. Without additional testing for those with suspected UTIs, it is not possible to confirm the appropriateness of prescribing and, therefore, difficult to assess possible implications on AMR. Additional work is needed around prescribing and testing and the capture of testing results to better assess prescribing practice and appropriate use.

## supplementary material

10.1136/bmjopen-2024-084485 online supplemental file 1

## Data Availability

Data may be obtained from a third party and are not publicly available.
