# Supplementary material for: Trends in urine sampling rates of general practice patients with suspected lower urinary tract infections in England, 2015–2022: a population-based study
Source: BMJ Open. 2024 Aug 6;14(8):e084485. doi: 10.1136/bmjopen-2024-084485 (PMC11308898; doi:10.1136/bmjopen-2024-084485)
Supplement: online supplemental file 1 [file bmjopen-14-8-s001.pdf]

Supplementary data

Figure S1- UTI episode linking

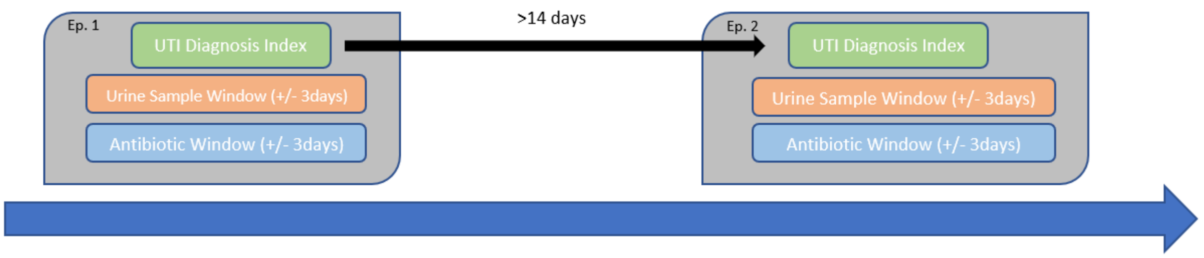

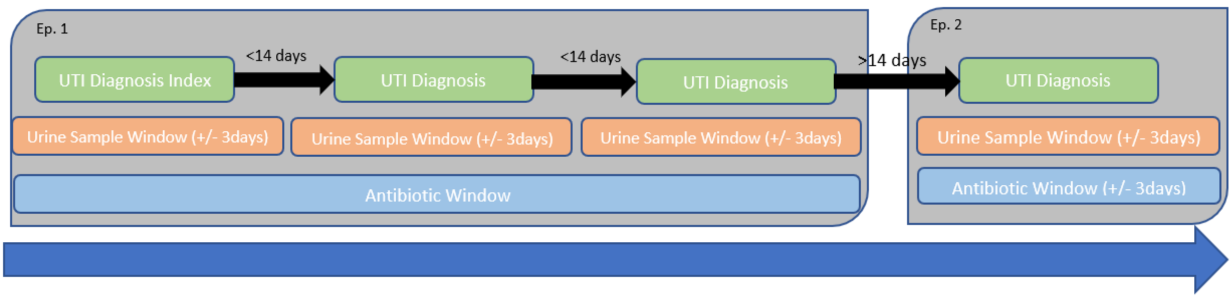

Figure S2- Percentage of episodes with urine testing and same-day UTI antibiotics

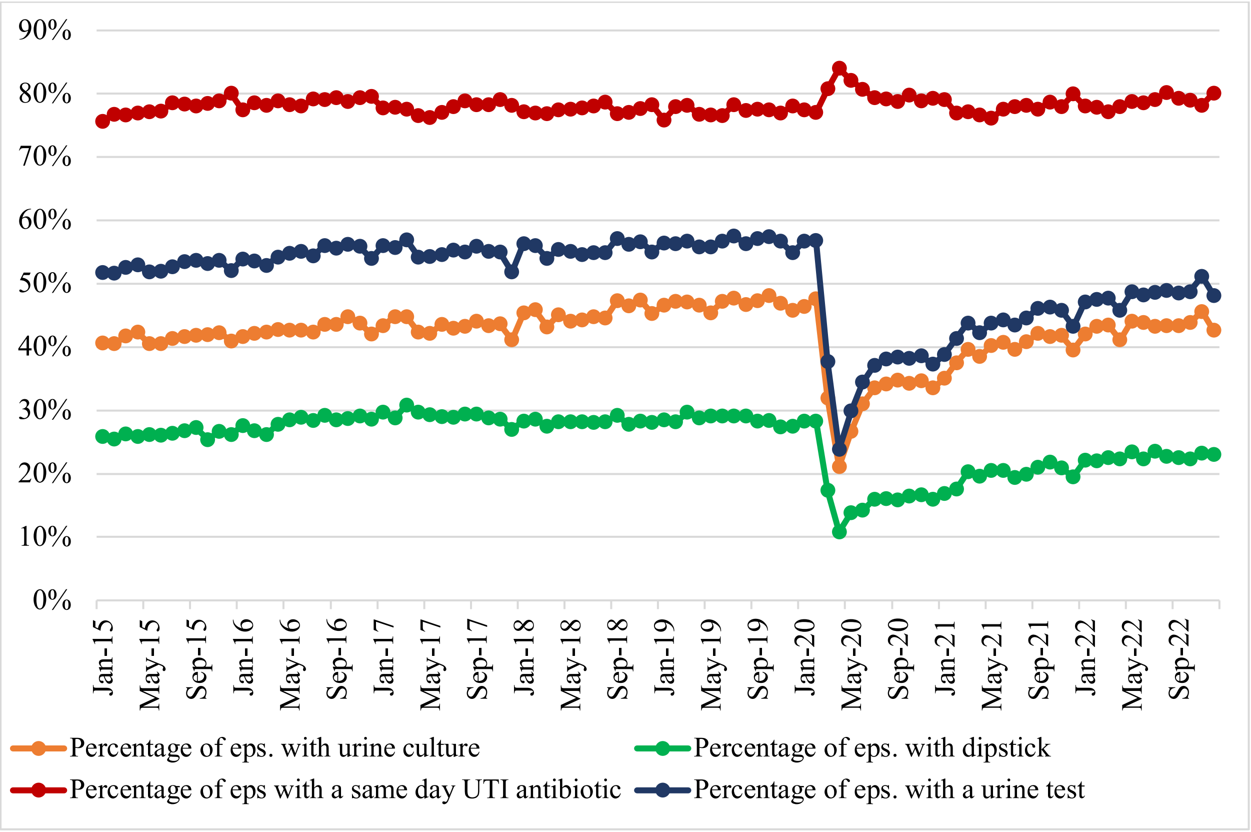

Table S1- Associations between episode factors and the odds of a urine test from binary logistic regression for those with and without a same-day antibiotic prescription

Variable	Category	Frequency	Univariate OR (95% CI)	p-value	Multivariate OR (95% CI)	p-value
With a Same Day UTI Antibiotic						
Age group	<16	35,934	1.93 (1.88, 1.97)	<0.001	1.75 (1.71, 1.79)	<0.001
	16-44	198,240	REF	-	-	-
	45-54	60,282	1.17 (1.15, 1.19)	<0.001	1.14 (1.12, 1.16)	<0.001
	55-64	63,331	1.29 (1.27, 1.31)	<0.001	1.25 (1.23, 1.27)	<0.001
	65-74	73,990	1.47 (1.45, 1.50)	<0.001	1.39 (1.37, 1.42)	<0.001
	75-84	82,815	1.31 (1.28, 1.33)	<0.001	1.25 (1.23, 1.27)	<0.001
	85+	64,522	0.89 (0.88, 0.91)	<0.001	0.86 (0.84, 0.87)	<0.001
Sex	Female	494,405	REF	-	-	-
	Male	84,709	1.27 (1.26, 1.30)	<0.001	1.75 (1.69, 1.81)	<0.001
Deprivation status	1	59,751	REF	-	-	-
	2	74,703	0.96 (0.94, 0.99)	<0.001	1.09 (1.07, 1.12)	<0.001
	3	64,017	1.01 (0.98, 1.09)	0.63	1.18 (1.15, 1.20)	<0.001
	4	61,548	1.07 (1.04, 1.09)	<0.001	1.25 (1.22, 1.28)	<0.001
	5	61,548	1.09 (1.06, 1.11)	<0.001	1.26 (1.23, 1.29)	<0.001
	6	45,833	1.24 (1.21, 1.27)	<0.001	1.40 (1.36, 1.44)	<0.001
	7	55,980	1.23 (1.20, 1.26)	<0.001	1.39 (1.35, 1.42)	<0.001
	8	55,910	1.36 (1.33, 1.40)	<0.001	1.51 (1.48, 1.55)	<0.001
	9	44,060	1.25 (1.22, 1.28)	<0.001	1.41 (1.37, 1.44)	<0.001
	10	63,119	1.20 (1.17, 1.22)	<0.001	1.33 (1.30, 1.36)	<0.001
Remote or face-to-face	Face to Face	325,964	REF	-	-	-
	Remote	229,056	0.39 (0.38, 0.39)	<0.001	0.42 (0.41, 0.42)	<0.001
	Other	11,694	0.68 (0.65, 0.70)	<0.001	0.67 (0.65, 0.70)	<0.001
Region	South	202,178	REF	-	-	-
	London	79,272	0.72 (0.70, 0.73)	<0.001	0.77 (0.76, 0.79)	<0.001
	Midlands	177,092	0.97 (0.96, 0.98)	<0.001	0.90 (0.89, 0.91)	<0.001
	North	120,572	1.02 (1.00, 1.03)	0.01	0.98 (0.96, 0.99)	0.01
Year	2015	72,655	REF	-	-	-
	2016	76,207	1.09 (1.07, 1.11)	<0.001	1.10 (1.08, 1.12)	<0.001
	2017	74,562	1.11 (1.09, 1.14)	<0.001	1.13 (1.11, 1.16)	<0.001
	2018	74,090	1.14 (1.12, 1.17)	<0.001	1.16 (1.14, 1.19)	<0.001
	2019	71,990	1.18 (1.13, 1.20)	<0.001	1.21 (1.19, 1.24)	<0.001
	2020	75,539	0.51 (0.50, 0.52)	<0.001	0.73 (0.71, 0.74)	<0.001
	2021	68,925	0.63 (0.62, 0.65)	<0.001	0.94 (0.92, 0.96)	<0.001
	2022	65,146	0.79 (0.77, 0.80)	<0.001	1.06 (1.04, 1.09)	<0.001
Number of episodes per practice by quartile	1 (lowest)	144,723	REF	-	-	-
	2	142,233	1.10 (1.08, 1.12)	<0.001	1.23 (1.21, 1.35)	<0.001
	3	145,051	1.09 (1.07, 1.10)	<0.001	1.23 (1.20, 1.25)	<0.001
	4	147,107	0.99 (0.97, 1.00)	0.09	1.18 (1.15, 1.21)	<0.001
Practice size by quartiles	1 (lowest)	157,863	REF	-	-	-
	2	153,250	0.90 (0.89, 0.91)	<0.001	0.80 (0.78, 0.83)	<0.001
	3	142,481	0.88 (0.87, 0.89)	<0.001	0.80 (0.78, 0.82)	<0.001
	4	125,520	0.90 (0.88, 0.91)	<0.001	0.84 (0.82, 0.86)	<0.001

With no Same Day Antibiotic						
Age group	<16	15,433	REF	-	-	-
	16-44	46,728	1.26 (1.22, 1.31)	<0.001	1.21 (1.16, 1.25)	<0.001
	45-54	14,167	1.29 (1.24, 1.31)	<0.001	1.19 (1.14, 1.23)	<0.001
	55-64	16,247	1.36 (1.31, 1.41)	<0.001	1.19 (1.15, 1.24)	<0.001
	65-74	20,528	1.34 (1.29, 1.38)	<0.001	1.15 (1.11, 1.19)	<0.001
	75-84	26,042	1.15 (1.12, 1.19)	<0.001	1.00 (0.96, 1.03)	0.84
	85+	22,445	0.88 (0.85, 0.91)	<0.001	0.76 (0.73, 0.79)	<0.001
Sex	Female	123,078	REF	-	-	-
	Male	38,512	0.87 (0.85, 0.89)	<0.001	1.15 (1.12, 1.18)	
Deprivation status	1	16,873	REF	-	-	-
	2	20,461	0.96 (0.92, 1.00)	0.07	1.12 (1.08, 1.17)	<0.001
	3	18,232	0.99 (0.95, 1.03)	0.68	1.17 (1.12, 1.23)	<0.001
	4	17,980	1.18 (1.13, 1.23)	<0.001	1.34 (1.28, 1.40)	<0.001
	5	15,094	1.11 (1.06, 1.15)	<0.001	1.25 (1.20, 1.32)	<0.001
	6	12,293	1.34 (1.28, 1.40)	<0.001	1.41 (1.34, 1.49)	<0.001
	7	15,208	1.40 (1.34, 1.46)	<0.001	1.40 (1.33, 1.46)	<0.001
	8	15,966	1.46 (1.40, .153)	<0.001	1.46 (1.39, 1.53)	<0.001
	9	12,307	1.31 (1.25, 1.37)	<0.001	1.33 (1.27, 1.40)	<0.001
	10	16,747	1.29 (1.24, 1.35)	<0.001	1.30 (1.24, 1.36)	<0.001
Remote or face-to-face	Face to Face	85,171	REF	-	-	-
	Remote	51,570	0.66 (0.65, 0.68)	<0.001	0.64 (0.62, 0.65)	<0.001
	Other	20,389	0.20 (0.19, 0.21)	<0.001	0.21 (0.20, 0.21)	<0.001
Region	South	59,834	REF	-	-	-
	London	23,715	0.55 (0.53, 0.57)	<0.001	0.66 (0.64, 0.69)	<0.001
	Midlands	45,618	0.96 (0.94, 0.98)	0.001	0.97 (0.94, 1.00)	0.02
	North	32,423	1.15 (1.12, 1.18)	<0.001	1.11 (1.08, 1.15)	<0.001
Year	2015	20,747	REF	-	-	-
	2016	20,596	1.07 (1.03, 1.12)	<0.001	1.09 (1.05, 1.14)	<0.001
	2017	21,252	1.06 (1.02, 1.10)	0.004	1.15 (1.10, 1.19)	<0.001
	2018	21,479	1.07 (1.03, 1.11)	<0.001	1.15 (1.10, 1.19)	<0.001
	2019	21,115	1.15 (1.10, 1.19)	<0.001	1.27 (1.22, 1.32)	<0.001
	2020	19,188	0.89 (0.86, 0.93)	<0.001	1.09 (1.04, 1.13)	<0.001
	2021	19,595	0.99 (0.95, 1.03)	0.70	1.23 (1.18, 1.28)	<0.001
	2022	17,618	1.09 (1.05, 1.13)	<0.001	1.28 (1.23, 1.34)	<0.001
Number of episodes per practice by quartile	1 (lowest)	38,971	REF	-	-	-
	2	40,122	1.26 (1.23, 1.30)	<0.001	1.34 (1.29, 1.38)	<0.001
	3	44,511	1.37 (1.34, 1.41)	<0.001	1.47 (1.42, 1.53)	<0.001
	4	37,986	1.32 (1.28, 1.35)	<0.001	1.38 (1.32, 1.45)	<0.001
Practice size by quartiles	1 (lowest)	42,656	REF	-	-	-
	2	46,677	0.95 (0.92, 0.97)	<0.001	0.80 (0.77, 0.82)	<0.001
	3	40,112	0.86 (0.83, 0.88)	<0.001	0.67 (0.64, 0.70)	<0.001
	4	32,145	1.00 (0.97, 1.03)	0.98	0.72 (0.68, 0.75)	<0.001
