# Supplementary material for: Trends in urine sampling rates of general practice patients with suspected lower urinary tract infections in England, 2015–2022: a population-based study
Source: BMJ Open. 2024 Aug 6;14(8):e084485. doi: 10.1136/bmjopen-2024-084485 (PMC11308898; doi:10.1136/bmjopen-2024-084485)
Supplement: Supplementary file 2 [file bmjopen-2024-084485.reviewer_comments.pdf]

PEER REVIEW HISTORY

BMJ Open publishes all reviews undertaken for accepted manuscripts. Reviewers are asked to complete a checklist review form (**http://bmjopen.bmj.com/site/about/resources/checklist.pdf**) and are provided with free text boxes to elaborate on their assessment. These free text comments are reproduced below.

ARTICLE DETAILS

**TITLE (PROVISIONAL)**	Trends in urine sampling rates of general practice patients with suspected lower urinary tract infections in England, 2015-2022: a population-based study
**AUTHORS**	Ciaccio, Laura; Fountain, Holly; Beech, Elizabeth; Brown, Colin S; Demirjian, Alicia; Gerver, Sarah; Muller-Pebody, Berit; Bou-Antoun, Sabine

VERSION 1 – REVIEW

**REVIEWER**	Llor, Carl<br>University of Southern Denmark, Department of Public Health, General Practice.
**REVIEW RETURNED**	26-Jan-2024

**GENERAL COMMENTS**	The paper is well-written, and the results are expected, except for a reduction in urine tests ordered for patients aged 85 and older. This study has some limitations, inherent to the studies which use research databases, but they are correctly addressed in the Discussion section.<br><br>I miss a better discussion of your results and compare them with other studies. Why do you think that fewer urine tests are ordered in the most elderly population?<br><br>The introduction could be shortened and be more focused to the aim of the paper.<br><br>You should make more clear when urine tests are necessary based on the UK recommendations. A high percentage of urine tests are ordered in otherwise healthy non-pregnant women. You should better discuss this 'overtesting' in your paper.<br><br>Are you specifically examining lower urinary tract infections (UTIs)? Did you differentiate between lower and upper UTIs in your study? Make this clearer please.

**REVIEWER**	Laan, Bart<br>Amsterdam UMC - Locatie AMC, Internal Medicine, Infectious Diseases
**REVIEW RETURNED**	10-Mar-2024

**GENERAL COMMENTS**	This manuscript provides the outcome of big data about urine testing in primary care in England. This study highlights a change in practice during the COVID pandemic, with less testing and more remote consultations for an antibiotic prescription. However, I

	would be helpful if the authors could clarify what their study adds to previous studies.<br><br>-It is unclear what the research question is.<br>-Why is only a subset of patients from GP practices used? How many are excluded?<br>-Did the advised testing strategy changed in the 2018 NICE UTI guideline update?<br>-Why are there only urine cultures and dipsticks rates? Are there no other urine test performed by the GP?<br>-Why is the rate of patients with a dipstick performed only 25%? Are the patients with a positive test the ones that are treated with antibiotics?<br>-How many patients are treated with antibiotics without positive urine test?<br>-The authors clearly describe all results and time trends during the years, but I miss some interpretation and reasons for presentation of all these numbers/rates. It would be of more value to know how many urine test weren't performed with good indication and how many are inappropriately performed without indication? What rate of antibiotics are overuse?<br>-It is a important limitation that data is limited to GP records. Urinalysis could be missed and therefore conclusions could be biased. Further, there is no data about patient symptoms. Since rates of ASB are high this is also a limitation.

**REVIEWER**	Albrecht, Uwe<br>Mediconomics GmbH
**REVIEW RETURNED**	13-Mar-2024

**GENERAL COMMENTS**	Recommendation:<br>Accept.<br>Manuscript ID: bmjopen-2024-084485<br>Title: Urine sampling rates of general practice patients in England, 2015-2022<br>The manuscript submitted showed a generation of data in an interesting area of the need for antibiotic treatment in patients with urinary tract infections. The patient population would benefit tremendously if a therapeutic need for standard antibiotic treatment could be clarified quickly, also with regard to the development of antibiotic resistance. The authors of the paper showed an overview of the respective areas in England and thus obtain a suitable first information in established practices, which should usually be the point of contact in this disease field. Thanks to the authors and more data is needed in future.<br>One little issues which I like the authors to address:<br>i) Line 316: Should be “Supplemental Table 1”. There is no Table 2, right?

VERSION 1 – AUTHOR RESPONSE

Reviewer: 1

6. The paper is well-written, and the results are expected, except for a reduction in urine tests ordered for patients aged 85 and older. This study has some limitations, inherent to the studies which use

research databases, but they are correctly addressed in the Discussion section.

Response: Thank you for your feedback and for taking the time to review and comment on our work.

7. I miss a better discussion of your results and compare them with other studies. Why do you think that fewer urine tests are ordered in the most elderly population?

Response: Thank you for your feedback. We have added additional discussion comparing our results with those of other studies and addressing why we might see lower testing rates in elderly populations (lines 467-511, 426-435) .

8. The introduction could be shortened and be more focused to the aim of the paper.

Response: Thank you for your comment. We have removed some text from the introduction and added additional details around the paper's aim. We have clarified that this work aimed to assess changes in trends of urine sampling practice and antibiotic prescribing for patients with a suspected lower UTI in the context of the 2018 NICE guidelines and the COVID-19 pandemic (lines 119-126).

9. You should make more clear when urine tests are necessary based on the UK recommendations. A high percentage of urine tests are ordered in otherwise healthy non-pregnant women. You should better discuss this 'overtesting' in your paper.

Response: Thank you for your feedback. We have updated the text to make clear when testing is recommended, including for pregnant women, men, or children under 16, based on NICE guidance (lines 95-105). As we were not fully able to identify pregnant women or other health conditions in this study, it is difficult to confirm overtesting in women. However, we have updated language in the discussion to indicate that the rates seen in this study are high, potentially suggesting overtesting (lines 437-443).

10. Are you specifically examining lower urinary tract infections (UTIs)? Did you differentiate between lower and upper UTIs in your study? Make this clearer please.

Response: Thank you for your feedback. We have updated the study and text throughout to make it clear that we have focused on lower UTIs.

Reviewer: 2

11. This manuscript provides the outcome of big data about urine testing in primary care in England. This study highlights a change in practice during the COVID pandemic, with less testing and more remote consultations for an antibiotic prescription. However, I would be helpful if the authors could clarify what their study adds to previous studies.

Response: Thank you for your feedback and for taking the time to review. We have added additional text to the discussion to clarify that we believe this paper adds additional data around testing and prescribing over a longer time frame and is the first broad study on this topic after the publication of the 2018 NICE guidelines, and that we are able to build directly upon previous work from 2011 to 2015 (lines 360-366).

12. It is unclear what the research question is.

Response: Thank you for your comment. We have updated the introduction to make it clearer that this

study aims to examine the occurrence of urine sampling and antibiotic prescribing in the context of the 2018 NICE guidelines, as well as the changes brought on by the COVID-19 pandemic (lines 119-126).

13. Why is only a subset of patients from GP practices used? How many are excluded?

Response: Thank you for your question. A subset of GP practices was used as this was the data available from IQVIA as part of the IMRD. According to IQVIA, data is collected from GP practices using compatible practice management systems, including EMIS Health or Vision and contributing to THIN, A Cegedim Database, licensed by IQVIA. We have updated the language in the manuscript, lines 130-136.

14. Did the advised testing strategy changed in the 2018 NICE UTI guideline update?

Response: We have updated the language to clarify that the 2018 NICE guideline was the first formal guidance put in place for UTI treatment and management, with adults included. We have added context around the prior NICE Quality Standard published in 2015 and its recommendations for UTI management (lines 416-422).

15. Why are there only urine cultures and dipsticks rates? Are there no other urine test performed by the GP?

Response: Thank you for your questions. These were the tests of interest pulled from the NICE guidance and are the standard tests completed in UK general practice.

16. Why is the rate of patients with a dipstick performed only 25%?

Response: Thank you for your question. This lower rate is likely due to guidance around dipstick use, particularly in older adults, and advice around asymptomatic UTIs. We have added additional text about this to the discussion (lines 425-435).

17. Are the patients with a positive test the ones that are treated with antibiotics? How many patients are treated with antibiotics without positive urine test?

Response: Thank you for your questions. We have added additional data around episodes with positive test results and same-day UTI antibiotic prescribing. Across the study period, 23.7% of those with a same-day UTI antibiotic had a positive urine test result recorded with 81.3% of those with a positive urine test having a same-day UTI antibiotic (lines 286-290).

18. The authors clearly describe all results and time trends during the years, but I miss some interpretation and reasons for presentation of all these numbers/rates. It would be of more value to know how many urine test weren't performed with good indication and how many are inappropriately performed without indication? What rate of antibiotics are overuse?

Response: Thank you for your feedback. Based on the data used for this work, we are unable to say if antibiotics were overused or if testing was performed without indication, as only episodes with a UTI diagnosis code were included. While we can say how many episodes received a prescription without a urine test, we cannot say for certain there were no other indications or diagnoses for which the antibiotics were prescribed. For this work, our study cohort was those with a UTI diagnosis code captured, including suspected UTI. Testing and prescribing were then reviewed and examined using the patient’s age and gender based on the guidance. We have tried to limit prescribing that may have been for other indications by limiting antibiotic prescriptions to those that occurred on the same day as

a UTI diagnosis code and by examining common UTI antibiotics outlined in the NICE guidelines. Still, we cannot be certain of other health factors that may have impacted prescribing. We have updated the text to provide more context around these numbers compared to those found in similar studies and comparing the testing and prescribing within the populations captured in this study (477-495).

19. It is a important limitation that data is limited to GP records. Urinalysis could be missed and therefore conclusions could be biased. Further, there is no data about patient symptoms. Since rates of ASB are high this is also a limitation.

Response: Thank you for your feedback. While this study aimed to examine sampling in general practice, we recognise that tests may have occurred outside of general practice. While some sampling may be missed in our data, we do not feel this harms the analysis based on the study's aim. We have, however, added additional text around this in the strengths and limitations section (lines 543-547). We have also acknowledged the likelihood of ASB, particularly in older populations, driving testing and positivity rates (lines 425-435).

Reviewer: 3

20. The manuscript submitted showed a generation of data in an interesting area of the need for antibiotic treatment in patients with urinary tract infections. The patient population would benefit tremendously if a therapeutic need for standard antibiotic treatment could be clarified quickly, also with regard to the development of antibiotic resistance. The authors of the paper showed an overview of the respective areas in England and thus obtain a suitable first information in established practices, which should usually be the point of contact in this disease field. Thanks to the authors and more data is needed in future.

Response: Thank you for your feedback and for taking the time to review our work.

21. One little issues which I like the authors to address:
i) Line 316: Should be “Supplemental Table 1”. There is no Table 2, right?

Response: Thank you for highlighting this error. This has been corrected.

In addition to addressing the above comments, we have corrected any additional errors noted during the review process along with other requested updates.

We look forward to hearing from you regarding our submission.

Sincerely,
Laura Ciaccio, on behalf of all authors
PhD Candidate, University of Dundee School of Medicine

VERSION 2 – REVIEW

**REVIEWER**	Llor, Carl<br>University of Southern Denmark, Department of Public Health, General Practice.
**REVIEW RETURNED**	10-Jun-2024

**GENERAL COMMENTS**	The authors have addressed and implemented my suggestions, and the paper is now improved.
